# A rapid review of emergency department interventions for children and young people presenting with suicidal ideation

**DOI:** 10.1192/bjo.2022.21

**Published:** 2022-03-04

**Authors:** Farazi Virk, Julie Waine, Clio Berry

**Affiliations:** Brighton and Sussex Medical School, University of Sussex, UK; Mental Health Liaison Team, Queen Alexandra Hospital, UK; Brighton and Sussex Medical School, University of Sussex, UK

**Keywords:** Suicide, suicidal ideation, management, emergency department, psychosocial interventions

## Abstract

**Background:**

Suicidal ideation is an increasingly common presentation to the paediatric emergency department. The presence of suicidal ideation is linked to acute psychiatric hospital admission and increased risk of suicide. The paediatric emergency department plays a critical role in reducing risk of suicide, strengthening protective factors and encouraging patient engagement with ongoing care.

**Aims:**

This rapid review aims to synthesise evidence on interventions that can be implemented in the paediatric emergency department for children and adolescents presenting with suicidal ideation.

**Method:**

Six electronic databases were searched for studies published since January 2010: PubMed, Web of Science, Medline, PsycINFO, CINAHL and Cochrane. Outcomes of interest included suicidal ideation, engagement with out-patient services, incidence of depressive symptoms, hopelessness, family empowerment, hospital admission and feasibility of interventions. The Cochrane risk-of-bias tool was used to evaluate the quality of studies.

**Results:**

Six studies of paediatric emergency department-initiated family-based (*n* = 4) and motivational interviewing interventions (*n* = 2) were narratively reviewed. The studies were mainly small and of varying quality. The evidence synthesis suggests that both types of intervention, when initiated by the paediatric emergency department, reduce suicidal ideation and improve patient engagement with out-patient services. Family-based interventions also showed a reduction in suicidality and improvement in family empowerment, hopelessness and depressive symptoms.

**Conclusions:**

Paediatric emergency department-initiated interventions are crucial to reduce suicidal ideation and risk of suicide, and to enhance ongoing engagement with out-patient services. Further research is needed; however, family-based and motivational interviewing interventions could be feasibly and effectively implemented in the paediatric emergency department setting.

The paediatric emergency department (PED) plays an integral role in ensuring children and adolescents at risk of suicide have timely access to appropriate resources. Suicide rates have increased in adolescents aged 15–19 years from 3.1 to 5.7/100 000 between 2010 and 2019 in the UK.^1^ Approximately 13% of 5- to 19-year-olds have at least one mental disorder;^2,3^ mental health presentations to a UK emergency care centre have increased threefold compared with 2019, and the most common reason for referral to Child and Adolescent Mental Health Services (CAMHS) in 13- to 17-year-olds was intentional overdose or self-harm.^3^ In 2018, there were 204 suicides recorded in England and Wales in young people aged 10–19 years.^4^ Suicide denotes ‘the act of intentionally ending one's life’.^5^ Mental health problems among children and young people appear to be increasing, as does suicidal ideation. Moreover, in early 2020, the COVID-19 pandemic began to place an additional significant burden on child mental health and have a substantial impact on psychosocial development.^6^ In Ireland, mental health attendances to the PED initially decreased by 26.8% during the first 4 months of the pandemic; by July and August, mental health presentations increased by 54.4% and 45.5% from September to December compared with 2019 data, highlighting the impact of COVID-19 on child mental health.^7^ Although the strongest predictor for suicide remains a previous suicide attempt, a third of adolescents who experience suicidal ideation for the first time go on to attempt suicide.^8,9^ Consequently, it is imperative to ensure that interventions offered to children and young people presenting to the PED are beneficial. Furthermore, the risk of a repeated suicide attempt is the highest during the first 6 months after a suicide attempt, which emphasises the importance of providing interventions that have a long-lasting effect, and of the need for robust follow-up post-discharge from the PED.^10,11^

A presentation of suicidal ideation has been considered as the most important sign of short-term suicide risk and warrants an in-depth clinical assessment.^5^ Studies have found that talking about suicide does not inadvertently create risk, and may lead to a reduction in distress in individuals who are experiencing suicidal thoughts.^12^ However, suicidal intent is difficult to measure, and a proportion of suicides occur as a result of individuals misjudging the risk.^5^ Children understand the concept of suicide and death as permanent by 8 years of age;^13^ nevertheless, clinicians must sensitively assess suicidal cognitions in children by in the context of rapport and empathy, within an open discussion centred around patient well-being. Worryingly, 25% of patients presenting to the PED who did not declare suicidal thoughts had suicidal ideation,^14^ and children and young people who died by suicide did not necessarily express recent suicidal ideation.^15^ Unrecognised suicidal ideation may be a result of insufficient time to explore patient well-being or a lack of mental health training for emergency department clinicians.^14^ The use of standardised screening tools is recommended.^16^ Results from a retrospective cohort study demonstrated that 53% of patients who presented to the PED with non-suicidal complaints were identified as having suicide risk when screened.^17^ Nonetheless, patients with an absence of suicidal ideation should not be deemed as having a lower risk of suicide.^5^ Self-harm is common in young people and engagement in these behaviours can be strongly linked to suicide.^18^ Self-harm refers to ‘intentional self-injury without wanting to die’, and frequently involves cutting, scratching, hitting and drug overdose.^19^ A UK study highlighted that 44% of deaths in individuals who presented to the hospital with non-fatal self-harm were attributed to suicide within the 10–18 years age group, over a 5-year follow-up period.^19^ Thus, children and adolescents presenting to the PED with suicidal ideation or self-harm should be considered at suicide risk.

## Factors affecting youth suicide

Effective suicide prevention strategies must be informed by the identification of factors that influence suicidality and youth suicide risk.^20,21^ Suicide occurs as a result of a combination of genetic, biological and psychosocial factors.^21^ Suicide risk in adolescence is decreased in the context of support provision, family stability, a network of friends, a positive school environment and economic security.^21^ Common aetiologies for suicidal ideation in the PED include physical and mental health problems, family instability and violence, bullying and school failure, trauma and bereavement, and otherwise insufficient access to resources that aid in the development of coping skills.^15,20,21^ Mental health problems are perhaps the most closely associated risk factor with suicidality.^22^ A cohort study found that children presenting with a combination of irritability, depressive and anxiety-related symptoms in childhood (age 6–12 years) were two times more likely to think about suicide or attempt suicide during adolescence (age 13–17 years), compared with those presenting with only irritability or depressive symptoms.^23^ This emphasises the importance of identifying symptoms in clinical settings and providing appropriate social and emotional support to children. Moreover, children with autism spectrum disorder and attention-deficit hyperactivity disorder are at a greater risk of depression and suicidal behaviour as they progress to adulthood.^24,25^

## Assessing and screening youth suicide risk

A universal screening tool has been proposed in a variety of medical settings, including the PED, primary care and school-based clinics. There are no standardised risk assessment tools used in the UK; however, the implementation of screening may be critical in reducing suicide, particularly for patients who do not disclose suicidal thoughts. The National Institute for Health and Care Excellence guidance advises clinicians to use the web-based tool ‘STOP’ to assess and monitor suicide risk in children.^16^ A study highlighted the benefits of using a suicide screening tool in the PED to help inform suicide prevention strategies. Ballard et al investigated the effectiveness of the Ask Suicide Screening Questionnaire on repeated PED visits.^26^ Results from the retrospective cohort study demonstrated that 53% of patients who presented to the PED with non-suicidal complaints also screened positive for suicide risk.^27,28^ Moreover, chronic childhood illnesses are significantly associated with depression in adulthood, and so addressing mental health presentations is important in reducing future suicide risk.^28^ Thus, screening tools that identify conditions such as autism spectrum disorder, attention-deficit hyperactivity disorder and chronic illnesses, may serve as an essential technique for assessing suicide risk and referral for emergency department interventions.^24^

Currently, patients presenting with suicidal ideation are reviewed, followed up or referred to out-patient services, depending on clinical judgement.^29^ Longer-term out-patient treatments include psychological interventions such as cognitive–behavioural therapy (CBT), family-based interventions and motivational interviewing.^30^ CBT is a goal-orientated therapy and involves collaboration between patients and psychotherapists to modify thought processes to facilitate change in mood.^31^ Family-based interventions focus on family dynamics and educating parents on signs of suicide, crisis planning and providing information on services.^32^ Motivational interviewing is centred on helping patients change their behaviours through listening and shared decision-making.^33^ However, implementing brief interventions in the PED, where patients are at high risk of suicide, may reduce short-term suicide risk and result in better engagement with out-patient follow-up.^29^ The World Health Organization recommends that brief interventions range from 5 min for brief advice to up to 30 min if including counselling.^34^ The Department of Health describes brief interventions as a vital approach for front-line workers to utilise with young people who may benefit from receiving information, and to aid in reducing harmful behaviours such as self-harm.^29^ Examples of brief interventions include informal discussions with youth, telephone services, one-to-one counselling within a youth programme and providing information in general practice or emergency department settings to reduce harm.^29^

## The current review

Previous systematic reviews have been conducted on youth suicide prevention in a variety of settings, yet further research is necessary.^35,36^ The current review aimed to improve upon the 2010 review by Newton et al by providing a new, up-to-date systematic search and synthesis in line with Preferred Reporting Items for Systematic Reviews and Meta-Analyses (PRISMA) guidelines,^35^ and to improve upon the 2018 review by Robinson et al^35^ by focusing on PED-specific interventions.^36^ Therefore, in this review, we aimed to evaluate findings from brief interventions as well as other strategies that could be adapted within the PED and be beneficial for managing suicidal ideation presentations. This review focuses on psychological intervention because of the rarity of primary research trials of pharmacological interventions with young people,^37^ and reported longer-term benefits of psychological interventions, including reducing the burden of ongoing mental health disorders into adulthood and improved quality of life, as highlighted in recent evidence.^38,39^ In addition, in light of the COVID-19 pandemic, mental health presentations to the PED are expected to continue exponentially, therefore a new review must be conducted to guide future suicide prevention.^6^ This review restricted focus to randomised controlled trials (RCTs) only, as they are considered to provide the strongest test of whether an intervention has an effect.^40,41^ By focusing on patients recruited from the PED, it may be possible to determine the factors associated with the success of specific interventions in this context.

This rapid review aimed to synthesise evidence on management interventions for children and adolescents presenting to the PED with suicidal ideation. Outcomes of interest included suicidal ideation, depressive symptoms, hopelessness, family empowerment, hospital admission, feasibility of the intervention and use of out-patient services and follow-up treatment to ascertain whether interventions improved suicidality.

The specific research questions were as follows: (a) what interventions have been used with children and adolescents presenting to the PED with suicidal ideation? and (b) what is the evidence for benefit of these interventions on suicidal ideation, associated mental health symptoms and engagement with out-patient services?

## Method

This rapid review^42,43^ was conducted in line with the PRISMA guidelines and conformed to the steps outlined in the 2009 PRISMA checklist.^44^ The protocol for this rapid review was pre-registered with International Prospective Register of Systematic Reviews (PROSPERO) on 3 February 2021 (reference number CRD42021225364). Ethical approval was not required because of the retrospective nature of the study.

### Search strategy

Six databases were searched on 17 December 2020: PubMed, Web of Science, Medline, PsycINFO, CINAHL and Cochrane. Other studies within the bibliography section of included studies were not searched. The following medical subject headings were used to screen titles, abstracts and keywords: ‘suicidal ideation’, ‘emergency department’, ‘children’, ‘adolescents’ and ‘management’. Search terms were combined using the Boolean operators ‘AND’ and ‘OR’. The search was restricted to articles published after January 2010. Filters including free full-text, publication date in the past 10 years and published in the English language were applied to the search results, and the full search is outlined in the Supplementary Material available at https://doi.org/10.1192/bjo.2022.21.

### Selection process

Articles were sought that reported an evaluation of any psychological/psychosocial/non-pharmacological intervention used with children or young people in the PED setting. Full inclusion and exclusion criteria are provided in Table 1.

**Table 1:** Population, Intervention, Comparison, Outcomes and Study (PICOS) inclusion and exclusion criteria

	Inclusion criteria	Exclusion criteria
Population	Children and adolescents aged 6–19 years At least 25% patients recruited from the paediatric emergency department	
Intervention	Psychological/psychosocial/non-pharmacological interventions targeting suicidality	Pharmacological interventions
Comparator	Any comparator, including treatment as usual	
Outcomes	Suicidal ideation, depressive symptoms, hopelessness, family empowerment and/or hospital admission And/or the feasibility of the intervention And/or out-patient services and follow-up treatment	
Study design	Randomised controlled trials Full text in the English language	Non-randomised controlled trials Non-English language Published before January 2010
Setting	Intervention deployed in clinical setting Any country	Interventions deployed outside clinical settings

Database search results were exported into the Mendeley software for screening (Mendeley Version 1.19.8 for Mac, Elsevier, Amsterdam, Netherlands; see https://www.mendeley.com/download-reference-manager/macOS) with the inclusion and exclusion criteria. The first author screened all records at title/abstract stage and full-text stage. The second author reviewed all full-text articles independently to determine the articles for final inclusion in the review.

### Data extraction

Data were extracted by the first author using a customised Microsoft Excel version 16.43 spreadsheet. The following data were extracted: study details, design, methods, participants, intervention and outcomes, including statistical significance. Study investigators were contacted for further information, clarification or missing information as necessary.

### Analysis

Because of the limited number of included studies and significant heterogenicity between outcome measures and intervention content, a full meta-analysis or sensitivity analysis was not appropriate. Therefore, studies were grouped by intervention and a range of outcome measures were analysed through narrative synthesis,^43,45^ using synthesis without meta-analysis guidelines.^46^

### Quality assessment

The Cochrane Risk-of-Bias Checklist (CRBT) for RCTs was used to evaluate the quality of the included studies.^47^ The studies were classified into ‘low risk of bias’, ‘high risk of bias’ or ‘unclear risk of bias’, using an algorithm generated by the CRBT tool that highlights features of the trial that are at risk of bias. The second author assessed the quality of studies independently.

## Results

### Study selection

**Fig. 1:**
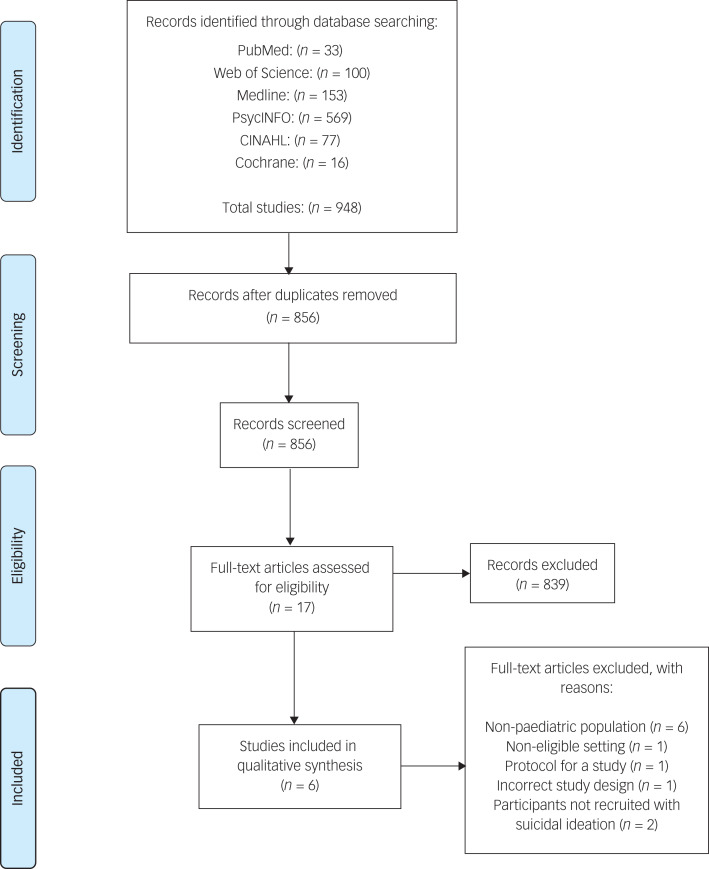
Preferred Reporting Items for Systematic Reviews and Meta-Analyses flow diagram detailing the screening and selection process.

The initial literature search yielded a total of 948 articles: 33 articles were published in PubMed, 100 in Web of Science, 153 in Medline, 569 in PsycINFO, 77 in CINAHL and 16 in Cochrane (see the PRISMA flow chart in Fig. 1). After duplicates were removed, 856 articles were screened at the title/abstract stage. At full-text stage, 17 articles were screened by the first and second author. A total of six articles met the criteria for final inclusion in the review.

### Study characteristics

The study characteristics for the six included studies^48–53^ are outlined in Table 2. All studies were published between 2010 and 2019. All included studies were conducted and published in the USA. Study sample sizes varied across all studies; the largest sample included 181 participants and the smallest sample included 49 participants. Participants were aged between 10 and 19 years. All studies took place in clinical settings^48,50–54^ recruited all participants in the PED setting. Participants were excluded from studies if they had signs of active psychosis, were requiring psychiatric hospital admission or had been recently discharged from hospital.

**Table 2:** Outlines the key characteristics of the included studies

Authors (year), country	Target population	Design	Participants	Intervention(s)	Control condition	Outcomes post-intervention	Outcomes at follow-up	Outcome measure, overall result and follow-up
Asarnow et al (2011), California, USA^53^Click or tap here to enter text.	Inclusion: ‘Presenting with suicide attempt and/or suicidal ideation.’ Exclusion: ‘Acute psychosis/symptoms impeding consent/assessment; no parent/guardian to consent, youth non English speaking, parents/guardians non English or Spanish speaking.’ Recruited from: PED	RCT	Sample *N* = 181, age range 10–18 years, treatment group *n* = 89, control group *n* = 92	FISP in emergency department designed to increase motivation for follow-up treatment and safety supplemented by telephone contacts after discharge. Delivered by: FISP clinicians. Clinicians with graduate mental health training received didactic training until certified proficient.	EUC: staff received one training session	Out-patient mental health treatment: FISP patients were significantly more likely than controls to be linked to out-patient treatment (92% *v*. 76%; odds ratio 6.2; 95% CI 1.8–21.3; *P* = 0.004) Suicidality: only reported at follow-up Depression: Not reported post-intervention	Out-patient mental health treatment: only reported post-intervention Suicidality: at follow-up, nine youths had attempted suicide (6%), four received the FISP intervention (6%) and five received enhanced usual emergency care (6%). One completed suicide Depression: statistically significant improvements from baseline to follow-up: CES-D total score (*t* = −8.5, d.f. = 130, *P* < 0.0001), severe CES-D (odds ratio 0.24, 95% CI 0.14–0.41, *P* < 0.0001)	Primary outcome: linking patients to out-patient mental health treatment and suicidality Exploratory outcomes: depression Overall: effective in linking youth to follow-up care and no statistically significant effect on suicidality Follow-up: 2 months
Diamond et al (2010), Philadelphia, USA^49^Click or tap here to enter text.	Inclusion: ‘Adolescents who scored >31 on the SIQ and above 20 on the BDI-II.’ Exclusion: ‘Adolescents needing psychiatric hospitalisation, recently discharged from a psychiatric hospital, current psychosis or mental retardation or history of borderline intellectual functioning.’ Recruited from: Primary care (75%) and PED (25%)	RCT	Sample *N* = 66, age range 12–17 years, treatment group *n* = 35, control group *n* = 31	ABFT: strengthening parent–adolescent bonds. Therapy starts by discussing what enables adolescents to turn to his/her parent(s) when contemplating suicide. Followed by a session for the adolescent to identify core family conflicts linked to suicide and prepares the adolescent to speak to his or her parent(s) in the next sessions. The next task focused on parental love, empathy and parenting skills. After this families came together to discuss identified problems and practice communication skills. The final task promoted adolescent autonomy while maintaining a family connection Delivered by: Not reported	EUC: a facilitated referral process with ongoing monitoring. Other providers set up initial appointments and encouraged participant attendance	Suicidal ideation: Not reported at post-intervention Depressive symptoms: Not reported at post-intervention	Suicidal ideation: 24 weeks, 82.1% of ABFT participants and 46.2% of EUC participants reported no suicidal ideation in the past week (odds ratio 5.37, 95% CI 1.56–18.49, *χ*2(1) = 7.66, *P* = 0.006) Depressive symptoms: 24 weeks follow-up, 58.1% of ABFT participants and 38.5% of EUC participants reported non-clinical depression scores (odds ratio 2.21, 95% CI 0.76–6.42, *χ*2(1) = 2.17, *P* = 0.14)	Primary outcomes: suicidal ideation and depressive symptoms Overall: ABFT showed a slightly higher rate of improvement for suicidal ideation. The intervention group showed significant improvements in depressive symptoms. The number of cases of suicidal ideation and repetition of self-harm was similar for both groups at the post-intervention period Follow-up: 6 months
Grupp-Phelan et al (2019), Ohio, USA^52^Click or tap here to enter text.	Inclusion: ‘Adolescents aged 12–17, positive screen for suicide risk on the ASQ tool, lived within 100 miles of the hospital/ had no contact with a mental health practitioner in the 90 days preceding emergency department visit and stable as determined by vital signs and triage criteria.’ Exclusion: ‘Chief concern of suicidal behaviour/primary or secondary psychiatric concern/altered mental status attributable to illness or medication, lacked telephone access, were unable to understand the study process or unable to speak/read English adequately to participate in study procedures.’ Recruited from: PED	RCT	Sample *N* = 168, age range 12–17 years, treatment group *n* = 84, control group *n* = 84	Brief motivational interviewing to target mental healthcare-seeking behaviour, barrier reduction discussion and referral. Participants received 1/2.5 follow-up telephone calls from the social worker who talked to the parent and assisted if problems arose with scheduling or accessing mental health treatment. Telephone contact was made within 2 days of discharge and before the scheduled appointment Delivered by: social workers who received a 2-day training by a Master's level certified motivational interviewing network trainer	EUC: brief mental health evaluation and referral following standard-of-care guidelines for emergency behavioural health assessments in emergency departments. Social workers did not receive new training. If the adolescent was safe to be discharged, a referral was made to a mental healthcare practitioner during a visit or the next day	Mental health treatment initiation: 2 months, the STAT-ED participants had similar rates of mental health treatment initiation compared with youth receiving EUC as assessed by parent report (29 [50.9%] *v*. 22 [34.9%]; adjusted odds ratio 2.08; 95% CI, 0.97–4.45) and administrative data from mental healthcare agencies (19 [29.7%] *v*. 11 [19.3%]; adjusted odds ratio 1.77; 95% CI, 0.76–4.15) Depression: (95% CI –4.8 to 3.7, *P* = 0.81). ‘There were no group or group × time effects in self-reported CES-D scores.’ Mean difference at 2 months: −0.28	Mental health treatment initiation: Overall rate and number of mental healthcare appointments for youth in the STAT-ED group were significantly higher at 6 months than for youth in the EUC group (mean, 3.25 [95% CI 1.89–4.62] *v*. 1.20 [95% CI 0.38–2.01]; *t*99.7 = 2.58; *P* = 0.01) Depression: (95% CI –4.0 to 6.6, *P* = 0.63). Mean difference at 6 months: 1.3	Primary outcomes: mental health treatment initiation and attendance within 2 months of emergency department discharge. Suicidal ideation and depression symptoms at 2 and 6 months. Exploratory outcomes: treatment initiation and attendance and suicide attempts at 6 months. Overall: no significant benefit on treatment initiation, attendance at 2 months and mental health outcomes at 2 and 6 months. In exploratory outcomes, STAT-ED outperformed EUC at 6 months in linking youth screening positive for suicide risk to initial and ongoing mental health treatment
Hughes and Asarnow (2013), California, USA^50^Click or tap here to enter text.	Inclusion: ‘Presenting to emergency department with a suicide attempt and/or suicidal ideation; aged 10 to 18.’ Exclusion: ‘No parent/guardian present to consent; acute psychotic symptoms or symptoms that would impair the ability to consent/complete assessments, non-English speaking youths, non-English or non-Spanish speaking parents.’ Recruited from: PED	RCT	Sample *N* = 181, age range 10–18 years, treatment group *n* = 89, control group *n* = 92	FISP delivered in the emergency department. A care linkage component with follow-up telephone contact to motivate and support linkage to out-patient treatment. Telephone contact: call within the first 48 h after emergency department or hospital discharge with additional contact at 1, 2 and 4 weeks Delivered by: FISP clinicians	Usual emergency department care enhanced by provider education	Suicidal ideation: the values for suicidal ideation were (95% CI −4.2 to 3.7, *P* = 0.90). Mean difference at 2 months: −0.24 Feasibility: Not reported at post-intervention	Suicidal ideation: (95% CI −3.3 to 8.3, *P* = 0.40). A significant decrease in suicidal ideation across the groups (*f* = 12.42, *P* ≤ 0.001) Mean difference at 6 months: 2.49 Feasibility: feasible and effective in linking youth to follow-up treatment	Primary outcomes: feasibility of FISP in emergency department and linking to out-patient treatment Overall:feasible and effective in linking youth to follow-up treatment Follow-up: 2 months
King at al (2015), Michigan, USA^51^Click or tap here to enter text.	Inclusion: ‘Being 14–19 years of age; having a positive suicide risk screen, defined as suicidal ideation, recent suicide attempt or positive screens for depression and alcohol or drug abuse. Presenting with a non-psychiatric chief complaint.’ Exclusion: ‘Level one trauma (critically ill/medically unstable), significant cognitive impairment (unable to complete self-report screen) or disposition of psychiatric hospitalisation.’ Recruited from: PED	RCT	Sample *N* = 49, age range 14–19 years, treatment group *n* = 27, control group *n* = 22	TOC: a crisis card with phone numbers for suicidal emergency support and written information. Personalised feedback about their screening responses. Participation in an adapted motivational interview (35–45 min) with a mental health professional. Adolescents received a follow-up note from their therapist 2–5 days after their visit, to support and facilitate the implementation of their plan Delivered by: study therapists completed 40 h of training conducted by member of the motivational interviewing network	EUC: a crisis card with phone numbers for suicidal emergency support and written information.	Suicidal ideation: Not reported at post-intervention Depression: Not reported at post-intervention Hopelessness: Not reported at post-intervention	Suicidal ideation: adolescents showed a decrease in suicidal ideation over the course of the study. (*d* = 0.22, *F* = 7.41, d.f. = 1.44, *P* < 0.01) TOC intervention showed large positive effects for depression (*d* = 1.07) and moderate positive effects for hopelessness at follow-up. (*d* = 0.40, *F* = 9.89, *P* = 0.01)	Primary outcomes: suicidal ideation, hopelessness, substance use and depression Overall: TOC intervention resulted in a greater reduction in depressive symptoms. TOC had a non-significant effect on suicidal ideation, both groups showed a significant reduction in suicidal ideation over the 2 months Follow-up: 2 months
Wharff et al (2017), Boston, USA^48^Click or tap here to enter text.	Inclusion: ‘Adolescents presenting to the emergency department with suicidality. Adolescents considered suicidal if, in the prior 72 h, they self-identified as suicidal, a parent/responsible adult noted behaviours indicating suicidality or the adolescent made a suicide attempt. Presence of a consenting parent or legal guardian with whom consent resides.’ Exclusion: ‘Either adolescent/parent lacked fluency in English, adolescent not medically stable including intoxication, adolescent demonstrated cognitive limitation prohibiting completion of research instruments, active psychosis, physical or medication restraint in the emergency department.’ Recruited from: PED	RCT	Sample *N* = 142, age range 13–18 years, treatment group *n* = 71, control group *n* = 68	FBCI received standard psychiatric evaluation and experimental intervention. A 60–90 min session helping to create a joint crisis narrative and taught cognitive–behavioural skill-building, therapeutic readiness, psychoeducation about depression and safety planning. The clinical team made recommendations for treatment with input from the patient and family. Delivered by: psychiatric social workers who were trained in the intervention	EUC: social workers did not receive new training. If the adolescent was safe to be discharged, a referral was made to a mental healthcare practitioner during a visit or the next day. TAU included standard psychiatric evaluation and clinic/discharge recommendations	Suicidality: no statistically significant change in RFL-A post-intervention Family empowerment: FBCI showed higher scores for FES compared with TAU (*F*_1,121_ = 8.1, *P* < 0.01)	Suicidality: no statistically significant change in RFL-A at 1-month follow-up.RFL-A scores increased over the study period indicating lower levels of suicidality. (*f* = 23.1, *P* < 0.001) Family empowerment: FBCI showed higher scores for FES compared with TAU at follow-up. Parents reported statistically increases in FES at 1-month follow-up. (*F*_4,431_ = 23.0, *P* = 0.005) Hospital admission: FBCI participants were less likely to be admitted to hospital compared with TAU (odds ratio, 3.4; 95% CI 1.7–6.8; *P* < 0.005)	Primary outcomes: presence and severity of adolescent suicidality, family empowerment, post-emergency department recommendation and disposition Secondary outcome: parent/guardian satisfaction and emergency department recidivism over the 1 month after the emergency department visit Overall: All participants reported lower levels of suicidality compared with baseline. FBCI group showed significantly higher scores for family empowerment and patient satisfaction. FBCI participants were significantly less likely to be admitted to hospital Follow-up: 3 months

PED, paediatric emergency department; RCT, randomised controlled trial; FISP, Family Intervention for Suicide Prevention; EUC, enhanced usual care; CES-D, Center for Epidemiological Studies for Depression; SIQ, Suicidal Ideation Questionnaire Junior; BDI-II, Beck Depression Inventory II; ABFT, attention-based family therapy; ASQ, Ask Suicide Screening Questions; STAT-ED, Suicidal Teens Accessing Treatment After an Emergency Department Visit; TOC, Teen Options for Change; FBCI, Family-Based Crisis Intervention; TAU, treatment as usual; RFL-A, Reasons for Living Inventory for Adolescents; FES, Family Empowerment Scale.

The studies evaluated family-based intervention (*n* = 4) and motivational interviewing (*n* = 2). Three family-based intervention studies conducted a brief intervention in the PED, followed by longer-term sessions post-discharge as therapy. One family-based intervention study conducted all stages of the RCT in the Department of Psychiatry at the Children's Hospital in Philadelphia. The motivational interviewing studies took place as brief interventions in the PED, including follow-up telephone calls post-discharge. Control conditions in the studies included provider education, a brief mental health referral, facilitated referrals, crisis cards and ongoing monitoring. Outcome measures differed between studies, and measures included a short-term risk of suicidal behaviour, motivation to seek follow-up treatment, suicidal ideation, depressive symptoms, family empowerment, hospital admission and feasibility of interventions. Study follow-up durations varied between 2, 3 and 6 months. Hughes and Asarnow^50^ did not comment on the study source of funding, but all other included studies were funded via health research grants.

### Study quality

**Fig. 2:**
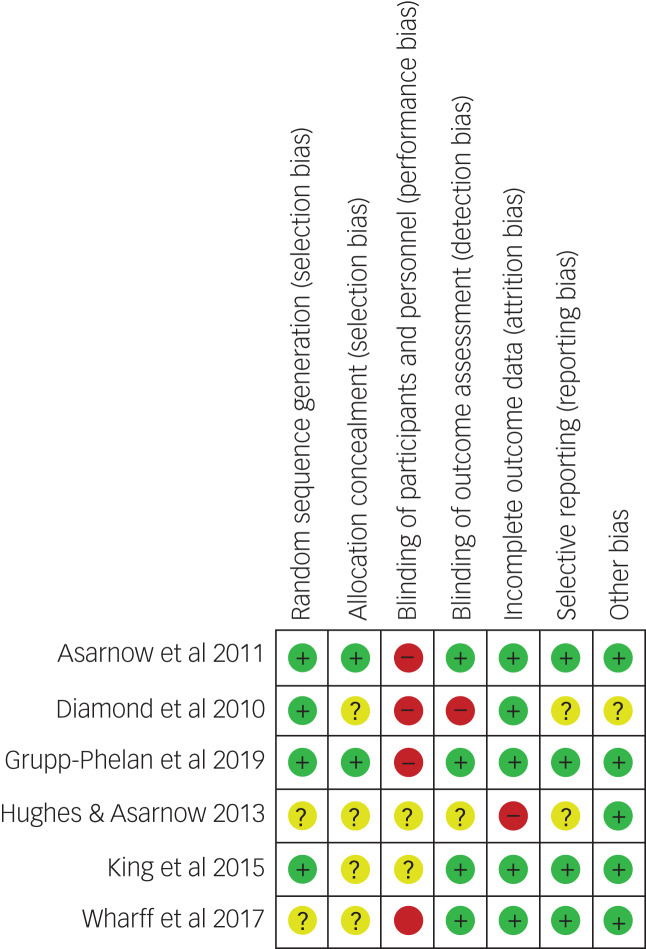
Summary of the risk of bias assessment with the Cochrane Risk-of-Bias Checklist.

The CRBT tool was used to assess the quality of included studies. Fig. 2 summarises the risk of bias assessments.^47^ Two studies were assessed as a low risk of bias.^47,48^ Two studies were assessed as unclear risk of bias because of the lack of information regarding randomisation, allocation concealment, blinding of outcome assessors and incomplete outcome data.^48,51^ Two studies were given a high risk of bias; one study had missing data without explanation.^44,45^

### Type of intervention

#### Family-based interventions

Four studies investigated the impact of family-based interventions on suicidal adolescents. The nature, content, duration, outcomes and follow-up period were variable across these four studies.^48–50,53^ Two studies explored the Family Intervention for Suicide Prevention (FISP) emergency department intervention, which included telephone contact post-discharge to motivate participants to engage with out-patient services.^50,53^ The FISP intervention by Asarnow et al involved a brief youth and family session in the PED focusing on educating families and developing a safety plan for future crises, delivered by clinicians with graduate mental health training who received didactic training with role playing.^53^ Following this session, structured telephone contacts were made to youth to motivate and support out-patient treatment within 48 h of discharge.^53^ Additional contacts were made at 1, 2 and 4 weeks post-discharge. Hughes and Asarnow designed the FISP intervention as a brief youth and family therapy session delivered in the emergency department.^50^ The full FISP intervention was delivered to 80.9% of participants in the emergency department; for youth discharged before completion of the FISP, the intervention was delivered on in-patient units after transfer from emergency department (12.4%), other community locations (3.4%) or via telephone (2.4%).^53^ Youth were discharged with a safety plan card with coping strategies and useful contacts.^53^ The intervention was delivered by clinicians with graduate training in psychology, social work, psychiatry or a related mental health field.^53^ Youth were contacted via telephone within 48 h of discharge, and additional contacts were made at 1, 2 and 4 weeks post-discharge.^53^ Diamond et al investigated an intervention, referred to as attention-based family therapy (ABFT), that focused on strengthening parent–adolescent bonds through face-to-face sessions, and was delivered by PhD or Master of Social Work level therapists who were trained by two of the study authors.^49^ Over six to eight sessions, participants completed five tasks that promoted family connectedness and adolescent autonomy.^49^ Parents were present for four to six sessions and adolescents completed two tasks alone.^49^ Wharff et al investigated the family-based crisis intervention (FBCI), designed to take place in the emergency department and delivered by trained psychiatric social workers.^48^ This involved psychiatric evaluation, including a 60–90 min session helping families with psychoeducation and safety planning.^48^ Participant follow-up durations differed between studies, ranging from 2 to 6 months.^48–50,53^

#### Motivational interviewing interventions

Two studies examined the effects of motivational interviewing on suicidal adolescents; Grupp-Phelan et al also explored the impact of motivational interviewing on treatment initiation and attendance within 2 months of discharge from the PED.^51,52^ The nature of motivational interviewing intervention differed between the two studies, but both interventions took place within the PED. The study by Grupp-Phelan et al involved four brief motivational interviewing sessions delivered to the adolescent and parent by trained social workers that targeted mental healthcare-seeking behaviour, barrier reduction discussion and referral.^52^ The adolescent and parent were interviewed alone for the first session; subsequent sessions involved the adolescent and parent together to discuss mental health options, potential barriers and next steps.^52^ After these sessions, participants received one or two follow-up telephone calls to discuss potential problems within their out-patient mental health treatment.^52^ Participants were followed up at 2 and 6 months.^52^ In the study by King et al, participants received a 35–40 min motivational interviewing session with a certified motivational interviewing mental health professional and a handwritten note from their therapist 2–5 days post-discharge; follow-up took place over 2 months.^51^ The study team also gave participants a crisis card for emergency suicidal support contacts and written information regarding depression, suicide risk, firearm safety and local mental health services.^51^

### Outcomes of interventions

#### Suicidal ideation

Five studies examined the impact of interventions on suicidal ideation, and outcome measures varied across studies.^48,49,51–53^ One study measured suicidal ideation with the Harkavy–Asnis Suicide Scale, to assess active and passive suicidal ideation.^53^ Three studies used the Suicidal Ideation Questionnaire-Junior to assess suicidal ideation.^48,51,52^ One study measured change over time in adolescent suicidality (Reasons for Living Inventory for Adolescents; RFL-A).^48^ Asarnow et al evaluated suicidality as an exploratory outcome; results illustrated no statistically significant intervention effects on suicidality.^53^ In the motivational interviewing intervention by Grupp-Phelan et al, there was a significant decrease in suicidal ideation across groups.^52^ Diamond et al found a slightly higher rate of improvement owing to a rapid reduction in suicidal ideation in the ABFT intervention group compared with the control group.^49^ At the end of the follow-up period, 82.1% of participants receiving the intervention reported no suicidal ideation in the past week compared with 46.2% of enhanced usual care (EUC) participants.^49^ Over the 6-month follow-up period, four out of 35 intervention group participants (11.4%) had made a suicide attempt, compared with seven out of 33 (21.2%) EUC participants.^49^ King et al reported a significant decrease in time for suicidal ideation over the study period.^51^ Wharff et al reported increases in the mean RFL-A total scores over the study period; however, there were no significant differences between the groups.^48^ This intervention illustrated that participants had lower levels of suicidality over time at 1-month follow-up compared with their baseline assessment.^48^

#### Depressive symptoms and hopelessness

Three studies explored the impact of the intervention on depressive symptoms.^49,51,52^ Diamond et al measured depression with the self-report Beck Depression Inventory, and results showed significant effects supported by large effect sizes.^49^ After treatment, at 6-month follow-up, 54.8% of ABFT participants and 31.0% of EUC participants had non-clinical depression scores.^49^ The Reynold Adolescent Depression Scale Short Form was used by King et al to measure depression; intervention (Teen Options for Change; TOC) participants demonstrated a significant positive change in depression, with a large effect size from baseline to follow-up.^51^ King et al also measured hopelessness with the Beck Hopelessness Scale, and results showed a moderate effect size for hopelessness.^51^ In contrast, Grupp-Phelan et al results showed no significant difference in depressive symptoms between intervention (Suicidal Teens Accessing Treatment after an Emergency Department Visit; STAT-ED) and EUC groups.^52^

#### Engagement with out-patient services

Two studies investigated the impact of interventions on engagement with out-patient services and treatment initiation.^52,53^ Grupp-Phelan et al explored treatment initiation and attendance. Exploratory outcomes showed no significant difference between the STAT-ED intervention and EUC in the rate of mental health appointments at 2-month follow-up.^52^ However, by 6 months, follow-up participants in the STAT-ED group were more likely to initiate mental health treatment and the overall rate of mental health appointments were significantly higher in the STAT-ED group compared with EUC.^52^ Asarnow et al intervention included a telephone contact within 48 h of discharge from the PED, to motivate and support out-patient treatment.^53^ More FISP participants were likely to receive out-patient treatment and had significantly more visits compared with the control.^53^

#### Family empowerment

In one study, family empowerment was measured as an outcome.^48^ Scores were obtained with a 34-item self-report Family Empowerment Scale (FES) that measures the level of empowerment of parents of a child with emotional difficulties.^48^ The FES questionnaire is completed by parents to assesses family, child and parental involvement within the community.^55^ Parents answer questions such as ‘I feel I am a good parent’, ‘I make sure I stay in regular contact with professionals who are providing my child services’ and ‘I have ideas about the ideal service system for children’.^55^ The scoring scale is rated 1–5; 1 equates to ‘never’ and 5 to ‘very often’.^55^ Wharff et al reported higher scores for family empowerment during the study.^48^ At the 1-month follow-up, there were statistically significant increases in the FES score.^48^

#### Hospital admission

One study evaluated the impact of the intervention on in-patient psychiatric hospital stay.^48^ The FBCI demonstrated that participants randomised to the intervention were significantly less likely to be admitted to hospital compared with treatment as usual.^48^ During the study, 68% of treatment-as-usual participants were admitted to hospital, compared with only 38% of FBCI participants.^48^

#### Feasibility

Hughes and Asarnow conducted a follow-up study of Asarnow et al to ascertain the feasibility of delivering FISP in the PED.^50,53^ Results showed that 80.9% received the intervention in the PED; however, because of discharge, FISP was delivered on in-patient units (12.4%), in the community (3.4%) or by phone (2.2%).^50^ In addition, 78.7% of FISP sessions were delivered with a parent and youth; however, 16.9% of FISP sessions were conducted with youth only, as some youth were brought to the PED by ambulance or police without their parents.^50^ Telephone calls were made to youth to enhance motivation and support for follow-up treatment at 48 h and 1, 2 and 4 weeks post-discharge; however, 88.8% of youth received at least one telephone call.^50^ This highlights potential barriers that become apparent after discharge, as successful contact with families requires clinicians and families to work together effectively. In addition, three participants withdrew from FBCI in the study by Wharff et al, and ten participants were lost to follow-up owing to being unable to reach by telephone.^48^ Similarly, four participants receiving TOC were discharged or left the hospital before motivational interviewing took place; three participants were lost to follow-up.^51^

## Discussion

This rapid review aimed to investigate interventions used in the PED setting for children and adolescents presenting with suicidal ideation. Six studies met the review inclusion criteria. All studies were initiated in the PED. The studies provided evidence for the impact of these interventions on suicidal ideation.^48–53^ Studies also outlined positive effects of interventions on patient engagement with out-patient follow-up treatment, depressive symptoms, hopelessness, family empowerment, hospital admission and intervention feasibility.^48–53^ To our knowledge, our study is the most recent and first rapid review to focus on a broad range of outcome measures to support PED care for young people presenting with suicidal ideation, as well as to identify areas requiring further research.

Two potential interventions were identified in this review; four studies involved family-based interventions and two studies comprised motivational interviewing interventions.^48–53^ Overall, findings suggest that family-based interventions are associated with a reduction in suicidal ideation, whereas evidence for the benefit of motivational interviewing is more equivocal. Overall, there is a lack of high-quality evidence because several limitations within the included studies, and therefore the conclusions should be drawn with caution.

Included studies that investigated the effects of family-based interventions on suicidal ideation consisted of dedicated sessions with families and patients in the PED to strengthen family bonds during a time of crisis. This is in keeping with a clinical review that highlighted early involvement of the family, formulation of risk evaluations and care based upon suicide risk and the availability of resources promote better outcomes.^56^ One study measured family empowerment and found statistically significant increases in the FES score;^48^ thus, it may be that the impact of family interventions is through the mechanism of empowering the family and mobilising family-based coping. Nonetheless, more high-quality studies investigating family-based interventions are required, with specific attention to the mechanisms of impact. However, a focus on family-based interventions must not detract from the importance of alternative intervention options in situations where family intervention may be inappropriate or unsafe; for example, for looked after children, or in the context of family conflict or domestic violence or abuse. Thus, it is important for the PED to be equipped with multiple intervention options and the skills to negotiate appropriate intervention provision, while retaining an atmosphere of collaborative patient care.

Furthermore, family-based interventions and motivational interviewing show some effect on depressive symptoms and hopelessness. Previous studies have^23^ suggested suicidality is linked to the experience of mental health problems such as depression.^22^ Moreover, hopelessness is implicated in suicidality, with greater hopelessness differentiating adolescents who attempt suicide from those with suicidal ideation but no attempts.^22^ However, further research is necessary to evaluate whether reductions in depression and hopelessness result in a reduction in suicidality.

An important component of suicide prevention is out-patient engagement, as studies have shown that patients who engage with services have a decreased risk of suicide.^57^ Two studies demonstrated that family-based interventions^50^ and motivational interviewing^52^ can increase out-patient treatment initiation and service use within the immediate 2 days after PED discharge^50^ and over the longer-term, i.e. 6 months after the intervention.^52^ In the study evaluating motivational interviewing, efforts were made in the intervention group to follow up on patients to check whether they were able to attend scheduled appointments, and telephone calls were made within 2 days post-discharge.^52^ The timing of follow-up contact has been highlighted as an important factor in managing suicide risk in patients who have been discharged after psychiatric hospital stay.^57^ A recent cohort study^58,59^ found that youth who had an out-patient mental health visit within 7 days after discharge had a decreased risk of suicide during the 6 months after psychiatric hospital stay.^57^ Thus, as a suicide prevention effort, contact must be made with patients within 7 days of discharge from any clinical setting.^57^

### Strengths and limitations

This novel rapid review has several strengths. First, the search of six high-yield databases facilitated a comprehensive search of relevant literature.^44^ Studies published within the past 10 years were included, which ensured that our conclusions were up to date. Only RCTs were eligible for this review; RCTs are considered the most valuable study methods for generating reliable high-quality data and assessing the effectiveness of interventions.^40,41^ The screening process was undertaken by one reviewer and the data extraction and quality assessment were checked by a second reviewer, to minimise bias during this process. Although the main outcome measure was a reduction in suicidal ideation, a broader set of outcomes were considered to ensure inclusion of additional factors associated with ongoing suicide risk and intervention implementation.

Some important limitations must nonetheless be borne in mind. Eligibility criteria were limited to studies published in the English language; broadening the criteria to non-English language studies may have resulted in additional studies, albeit their relevance to the UK healthcare system may be limited. Furthermore, this review yielded a small number of studies that displayed significant heterogeneity in interventions, outcomes and population. As such, a meta-analysis could not be performed because variations in interventions and outcomes, and primary research that has considerable risk of bias, may produce misleading or inappropriate meta-analytic results.^46^ Therefore, we made no pooled estimate of intervention effectiveness.

In addition, an important consideration is the exclusion of severe cases of suicidal ideation within reviewed studies; therefore, results may represent effects with young people presenting with less severe suicidality than seen in the PED generally. This reflects a broad tension in research trials around maximising the reach of an intervention (and research outcomes) to people potentially most at need of support as well as balancing safety concerns. Safety is an important consideration because psychological interventions may cause harm as well as give rise to benefits, and negative experiences of care immediately after events such as self-harm are seen to increase risk of further self-harm and hinder future disclosure.^60^ Current UK guidelines are to make an urgent referral to CAMHS for children and young people presenting with high risk of suicide (and depression), with the provision of a safe space to prevent injury as needed, and not to provide any psychological intervention *in situ.*^61^ Nonetheless, evidence for effective interventions that could be safely deployed in the PED for high-risk children and young people, in the context of the very high demand on CAMHS services,^62^ could build much-needed health service capacity and help to prevent deaths by suicide. The development of intervention protocols and evidence regarding intervention safety and effectiveness for adults and young people in the high-risk suicidality spectrum remains an important goal.^63^

All included studies were published and conducted in the USA; this highlights that the results of the review may not translate to the UK or other countries.^48–53^ Consequently, there are implications for the universal application of the interventions to other healthcare systems. For example, in the UK, mental health service funding is significantly limited; therefore, replicating the interventions in UK hospitals might be difficult.^64^ A literature review and thematic analysis of emergency department staff attitudes toward patients with a mental health problem highlighted that staff perceived caring for individuals with a mental health concern as a challenge and felt ill-prepared in assessing individuals.^65^ Therefore, this demonstrates that there is a lack of confidence in emergency department staff when approaching mental health presentations.

The studies reviewed were largely at high risk of bias. Many studies did not publish protocols or outline randomisation processes, and sample sizes were relatively small.^48–53^ Moreover, two studies^48,52^ recruited participants within restricted staff working hours, which was reported as office hours only in one study,^52^ and therefore the samples may not be representative of children presenting outside of usual office hours. Diamond et al recruited 75% of participants from primary care and 25% from the PED.^49^ This study did not disaggregate results for primary care and PED participants; there is a possibility that participants recruited through primary care differ in terms of initial presentation and response to intervention.^49^ Moreover, the eligibility criteria for participants in this review ranged from 6 to 19 years; however, the age of participants ranged from 10 to 19 years within included studies.^48–53^ Therefore, as the included studies did not test the intervention with children aged under 10 years, we could make no conclusions about the effectiveness and feasibility of delivering interventions in the PED in this group. However, children show signs of emotional and behavioural distress when exposed to parental conflict, and so it is important that family-based interventions are appropriate for all age groups and adapted for younger children.^66,67^

### Implications and priorities for future research

This review identified two interventions that demonstrated some improvements in suicidal ideation, with stronger evidence for the effectiveness of family-based interventions, especially regarding out-patient engagement. Currently, in the UK, patients requiring hospital admission are admitted as an in-patient; a child mental health liaison team within an acute hospital setting is rare, therefore patients are seen by a CAMHS professional the following day or they may wait several hours before seeing CAMHS within the PED. There is sufficient evidence to highlight the role of family as a protective factor against suicide; promoting cohesion and education of parents and children leads to better outcomes.^15^ Our results have shown promising approaches to family-based therapy, particularly ABFT.^49^ Based upon the literature supporting the importance of family–child relationships in suicidality onset and outcomes, we propose a family-based intervention within the PED and contact within 2 days post-discharge in a follow-up clinic.^16,44,58^ A priority must be to use a co-design process with children, young people, families and PED professionals to adapt interventions used in the USA for appropriate delivery in the UK PED setting. However, we acknowledge that some young people have difficult family relationships or do not have contact with parents or a guardian, such as looked after children, thus brief motivational interviewing may be an appropriate alternative.^56^ Although our review focused on managing suicidal ideation, we recommend training for emergency department staff in both being able to screen, assess and effectively identify young patients with suicidality,^68^ and in delivering brief psychological interventions in the PED setting.^29^ This would encourage patients to seek out-patient follow-up treatment, prevent readmission and keep costs minimal, which may aid in supporting community suicide prevention efforts.

This review has highlighted the lack of high-quality evidence to support the implementation of evidence-based interventions for youth suicidality in the PED setting. Thus, we recommend high-quality randomised trials with larger sample sizes, investigating and comparing family intervention and motivational interviewing approaches alongside other promising interventions. We recommend studies consider relevant subpopulations, including the evaluation of alternative interventions not involving family as relevant depending on family circumstances; for example, young people at very high risk of suicide, looked after children, and children with historical and/or current experiences of domestic violence and abuse. However, involving family where appropriate, by asking family empowerment questions within the PED to ascertain how families are coping, may result in better patient outcomes. We recommend performing cost-effectiveness analyses of potential interventions,^64^ to ensure intervention delivery would be cost-effective and sustainable. These recommendations would enable future systematic reviews and meta-analyses to be based upon more reliable studies.

Finally, despite the significant recent rises in suicide rates in young people generally and throughout the COVID-19 pandemic, there is limited high-quality evidence to illustrate the effectiveness of interventions. This review highlights the apparent benefits of psychological interventions delivered within the PED setting for children and young people presenting with suicidality, including improving mental health, depressive symptoms, hopelessness, family empowerment and hospital admission. Therefore, it is imperative to conduct more high-quality research to clarify definitive intervention outcomes. Studies must be undertaken within the UK specifically to establish successful emergency department-based interventions that can work effectively within this context.

## Data Availability

The data that support the findings of this study are available from the corresponding author, F.V., upon reasonable request.

## Supplementary material

For supplementary material accompanying this paper visit http://doi.org/10.1192/bjo.2022.21. click here to view supplementary material
