# Supplementary figures and images for: A rapid review of emergency department interventions for children and young people presenting with suicidal ideation
Source: BJPsych Open. 2022 Mar 4;8(2):e56. doi: 10.1192/bjo.2022.21 (PMC8935937; doi:10.1192/bjo.2022.21)

## Supplementary Files

## A1: Search strategy

PubMed Search


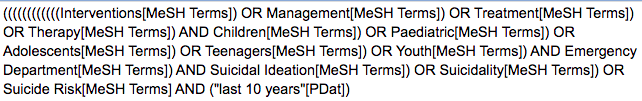

Supplement: Supplementary file 1 [file S2056472422000217sup001.docx]
